# Peril in the market-classification and dosage of species used as anti-diabetics in Lima, Peru

**DOI:** 10.1186/1746-4269-9-37

**Published:** 2013-05-30

**Authors:** Rainer W Bussmann, Narel Paniagua-Zambrana, Marinoli Rivas Chamorro, Natalia Molina Moreira, María Luisa del Rosario Cuadros Negri, Jose Olivera

**Affiliations:** 1 William L. Brown Center, Missouri Botanical Garden, PO Box 299, Saint Louis, MO 63166-0299, USA; 2 Herbario Nacional de Bolivia, Instituto de Ecología-UMSA, Campus Universitario, Cota Cota Calle 27, Apdo. correo central, La Paz, Postal 10077, Bolivia; 3 Museo de Historia Natural-UNMSM, Av Arenales 1256 Jesús María, Lima, Perú; 4 UEES (Universidad de Especialidades Espiritu Santo), Km 2.5 via Samborondon, Guayas, Ecuador; 5 Ministerio del Interior-Sanidad Policia Nacional, Hospital Geriatrico. Jr. Ramon Castilla Cuadra 5, Distrito de San Miguel, Lima, Perú; 6 CIBN, Facultad de Medicina San Fernando-UNMSM, Av Grau 755, Lima, Perú

**Keywords:** Medicinal plants, Taxonomy, Systematics, Falsification, Efficacy, *Gentianella*, *Geranium*, Gentianaceae, Geraniaceae, Andes, Plantas medicinales, Taxonomía, Sistematica, Falsificación, Eficacia, *Gentianella*, *Geranium*, Gentianaceae, Geraniaceae, Andes

## Abstract

**Background:**

Peru is what Peruvian anthropologist Lupe Camino calls the “health axis” of the old Central Andean culture area stretching from Ecuador to Bolivia. In particular in the North of the country the traditional use of medicinal dates back as far as the first millennium B.C. Both healers, and the wider population, often buy their medicinal plants in local markets, but there is very little comparative information available about which plants are sold under which vernacular name at any given time, for which indication, and which dosage information and information about side effects is given by vendors. For this study we used two traditionally used species groups “Hercampuri” *Gentianella* spec. (Gentianaceae) and “Pasuchaca” *Geranium* spec. (Geraniaceae*.*), found in the Mercado Aviación in Lima, as small, clearly circumscribed plant group frequently used to treat symptoms of diabetes as a test case to study the taxonomy, indications, dosage, indicated side effects, and additional species used as admixtures and hypothesized that: 1. A wide variety of different species is sold under the same common name, and often several common names exist for one species. 2. There is no consistency in the dosage, or a relationship between dosage and species marketed under one name. 3. However, there is consistency in the knowledge about usage and side effects.

**Methods:**

Surveys focusing on medicinal plants sold and their properties were conducted at the Mercado Aviación in Lima in December 2012. Vouchers of all specimens were deposited at the National Herbarium of Peru.

**Results and conclusions:**

Our surveys in Mercado Aviación in Lima yielded four species of *Gentianella*, two of *Geranium,* and three additional species from three genera used as common additives that were sold as anti-diabetic. These results indicate that even in case of only a few plant species, used for a very clearly circumscribed application, patients run a considerable risk when purchasing their remedies in the market. The possible side effects in this case are the more serious because diabetes has to be treated long term, and as such the patients are ingesting possible toxic remedies over a long period of time. Much more control, and a much more stringent identification of the material sold in public markets, and entering the global supply chain via internet sales, would be needed.

## Abstract (es)

**Resumen:**

**Antecedentes**

El Perú es lo que el antropólogo peruano Lupe Camino llama el “eje de la salud” de la vieja zona cultural andina central que se extiende desde Ecuador hasta Bolivia. En particular en el norte del país el uso tradicional de las fechas medicinales remonta hasta el primer milenio aC. Tanto los curanderos, como la población general de frecuencia compran sus plantas medicinales en los mercados locales. Hay poca información comparativa sobre cual plantas se venden bajo el mismo nombre vernacular, para que indicación, y que información de dosificación e información sobre los efectos secundarios proveen los vendedores. Para este estudio utilizamos dos grupos de espacies tradicionalmente usadas, “Hercampuri” *Gentianella* spec. (Gentianaceae) y “Pasuchaca” *Geranium* spec. (Geraniaceae*.*), vendidos en el Mercado Aviación en Lima, como un grupo pequeño de planta claramente circunscrito y utilizado con frecuencia para tratar los síntomas de la diabetes, como un caso de prueba para estudiar la taxonomía, los indicaciones, la dosificación, los efectos secundarios indicados y otras especies usados como aditivos bajo los hipótesis de que: 1. Una amplia variedad de especies diferentes se vende bajo el mismo nombre común, y a menudo existen varios nombres comunes para una especie. 2. No hay consistencia en la dosis, o una relación entre dosis y especies comercializadas bajo un mismo nombre. 2. Sin embargo, hay consistencia en el conocimiento en uso y los efectos lado.

**Métodos**

Las encuestas sobre las plantas medicinales vendidas y sus propiedades fueron realizadas en el Mercado Aviación en Lima en Diciembre de 2012. Muestras de todos los especímenes fueron depositados en el Herbario Nacional del Perú.

**Resultados y Conclusiones**

Nuestros estudios en Mercado Aviación en Lima encontraron cuatro especies de *Gentianella*, dos de *Geranium*, y tres especies de tres géneros usados como aditivos que fueron vendidos como antidiabético. Los resultados indican que, incluso en caso de sólo unas pocas especies de plantas utilizadas para una aplicación muy claramente delimitada, los pacientes corren un alto riesgo en la compra de sus remedios en el mercado. Los efectos secundarios posibles en este caso pueden ser más serio ya que la diabetes tiene que ser tratado a largo plazo, y como tal, los pacientes ingieren posibles remedios tóxicos durante un largo período de tiempo. Mucho más control y una identificación mucho más estricta del material vendido en los mercados públicos, y entrando en la cadena de suministro global a través de las ventas por Internet, sería necesario.

## Background

Northern Peru is what Peruvian anthropologist Lupe Camino calls the “health axis” of the old Central Andean culture area stretching from Ecuador to Bolivia [1]. In particular in the North of the country the traditional use of medicinal dates back as far as the first millennium B.C. (north coastal Cupisnique culture) or at least to the Moche period (100–800 AD), with healing scenes and healers frequently depicted in ceramics [2]. The extraordinary diversity of plant use in the region has always attracted scientific study. Early ethno botanically oriented studies focused mainly on the famous “magical” and “mind altering” flora of Peru. The first study on “*cimora*” -another vernacular name for the San Pedro cactus (*Echinopsis pachanoi*) dates back to the 1940’s [3]. The first detailed study on a hallucinogen in Peru focused also on San Pedro, and Tree Datura (*Brugmansia* spp.) [4-8]. Coca (*Erythroxylum coca*) also attracted early scientific attention [9-13], as did the Amazonian Ayahuasca (*Banisteriopsis caapi*) [14-16]. Chiappe et. al [17] were the first to attempt an overview on the use of hallucinogens in shamanistic practices in Peru. General ethno botanical studies in Peru and Bolivia focused mostly on Quechua herbalism of the Cusco area [18-22], the border region of Peru and Bolivia around Lake Titicaca [23-26] and the Amazon [27-29]. Northern Peru, and especially its large medicinal plant markets, was studied more recently [30-38].

Both healers, and the wider population, often buy their medicinal plants in local markets. Information on the composition of the market flora, the origin of the plant material, and the quantities of plant material sold exists to some extent [37,39-41], and some studies focus on the interface between traditional and allopathic medicine [42,43]. Peru occupies a middle rank (125 of 223 countries surveyed) in diabetes prevalence (5–6% of the population) [44], and the allopathic concept of diabetes is well known. Previous studies [33,34,42,43] indicate that patients regularly receive their diagnosis from allopathic doctors, and then go to the markets to buy traditional remedies. Vendors also followed the allopathic concept. Initial bioassays indicate that at least for antibacterial applications the efficacy of parts of the medicinal flora can be proven [45-52], and preliminary data on plant toxicity exist [53]. However, there is very little comparative information available about which plants are sold under which vernacular name at any given time, for which indication, and which dosage information, and what kind of information about side effects vendors give.

For this study we used two traditionally used species groups “Hercampuri” *Gentianella* spec. (Gentianaceae) and “Pasuchaca” *Geranium* spec. (Geraniaceae*.*), found in the Mercado Aviación in Lima, as small, clearly circumscribed plant group frequently used to treat symptoms of diabetes as a test case to study the taxonomy, indications, dosage, indicated side effects, and additional species used as admixtures and hypothesized that:

1. A wide variety of different species is sold under the same common name, and often several common names exist for one species.

2. There is no consistency in the dosage, or a relationship between dosage and species marketed under the same vernacular name.

3. There is consistency in knowledge on usage and side effects.

## Materials and methods

### Surveys

Surveys focusing on plants sold under the traditional species concepts “Hercampuri” (*Gentianella* sp.) and “Pasuchaca” (*Geranium* sp.) to treat diabetes, as well as species used as additives were conducted at the Mercado Aviación in Lima in December 2012. Researchers would approach the vendors and explain the premise for the study. From those who gave their prior informed consent, information was collected regarding their inventory of “Hercampuri” and “Pasuchaca”, as well as of additive species. The vendors were asked about other vernacular names, medicinal indications the plants were used for, dosage, side effects, admixtures to these species, and provenance and seasonality of the material.

### Voucher collection

The specimens are registered under the collection series “RBU,” “MRCH,” “MONA,” “JOG”. Vouchers of all specimens were deposited at the National Herbarium of Peru in the Museo de Historia Natural San Marcos (USM). In order to recognize Peru’s rights under the Convention on Biological Diversity, especially with regard to the conservation of genetic resources in the framework of a study on medicinal plants, the identification of the plant material was conducted entirely in Peru. No plant material was exported in any form whatsoever.

### Species identification and nomenclature

The nomenclature of plant families, genera, and species follows the Catalogue of the Flowering Plants and Gymnosperms of Peru [54]. The nomenclature was compared to the TROPICOS database (http://www.tropicos.org). Species were identified using the available volumes of the Flora of Peru [55], as well as [56-58], and reference material in the National Herbarium of Peru (USM). Species and author names for all species encountered are given in Additional file 1.

## Results and discussion

**Figure 1:**
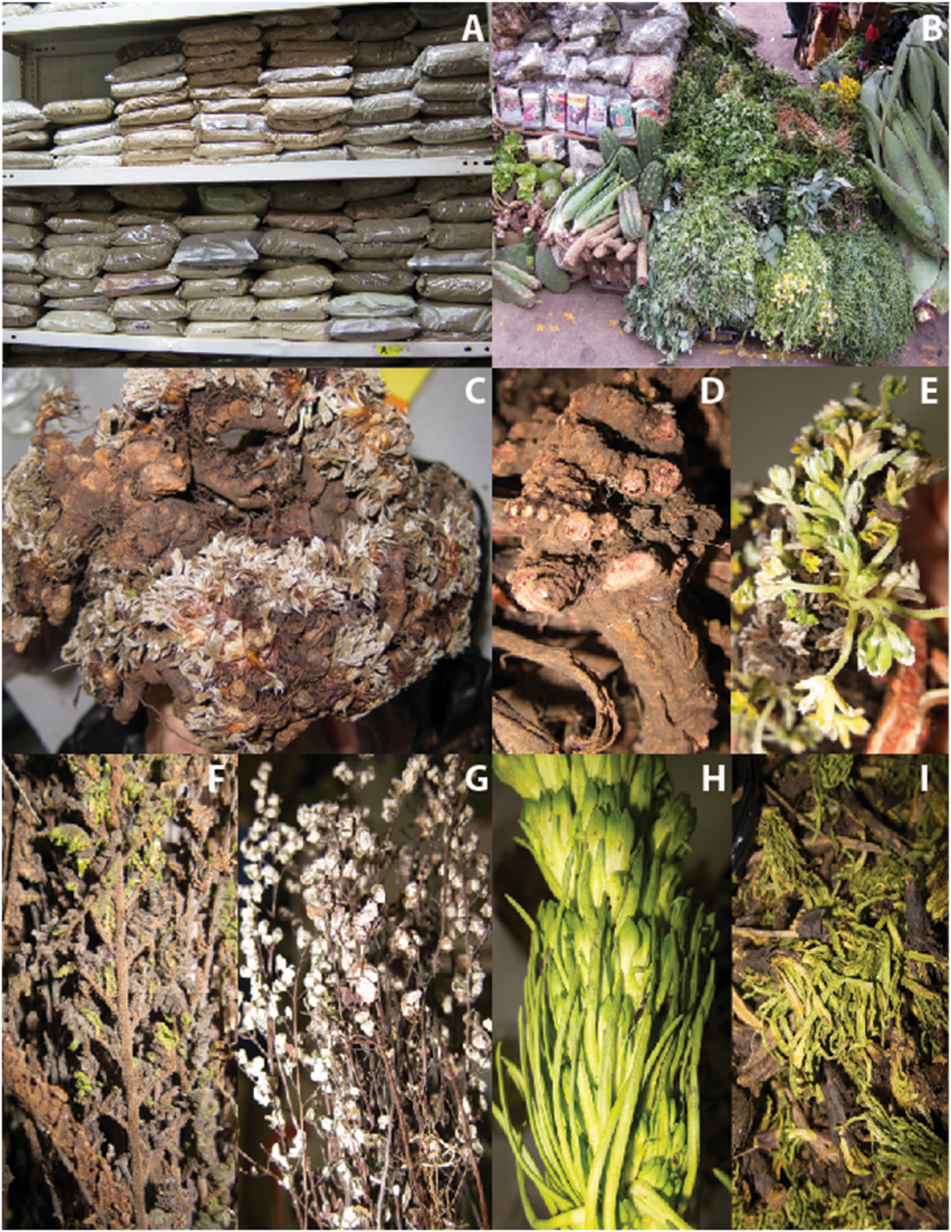
**Medicinal plant presentation in Mercado Aviación: A. packaged ground plat material; B. traditional presentation; C-E. *Geranium sessiliflorum*; D. unidentifiable *Geranium* fragment; F. *Cheilanthes bonariensis*; G. *Argyrochosma nivea; *H. *Gentianella thyrsoidea*; I. *Gentianella nitida***.

Our surveys in Mercado Aviación in Lima yielded four species of *Gentianella* sold as “Hercampuri”, two of *Geranium* sold as “Pasuchaca”, and three additional species from three genera used as common additives that were sold as anti-diabetic. *Gentianella nitida* (Grieseb.) Fabris and *Gentianella thyrsoidea* (Hook.) Fabris were the most commonly sold Gentianaceae, whereas only small samples of *Gentianella incurva* (Hook.) Fabris and *Gentianella tristicha* (Gilg.) J.S. Pringle could be found. All these species were sold as “Hercampuri”. Interestingly, not a single sample of *Gentianella alborosea* (Gilg) Fabris, more commonly mentioned in literature as “Hercampuri” was encountered. *Geranium sessiliflorum* Cav. and *Geranium crassipes* Hook. ex. Grey were both sold as “Pasuchaca”, and again, *Geranium dielsianum* Kunth., the only species mentioned in the phytomedical literature, was not encountered. *Argyrochosma nivea* (Poir.) Desv. and *Cheilanthes bonariensis* (Willd.) Proctor (both Pteridaceae) were interchangeably sold as “Cuti Cuti), and together with *Morus alba* L. (Moraceae) were used as admixtures in preparations to treat diabetes (Figure 1).

The species used, their indications, and the vernacular names under which they were sold, greatly differed not only from literature on similar markets in other parts of the country, but also from previous studies conducted in Lima (Additional file 1).

Albán [59], Lima, reported *Gentianella alborosea* (Gilg.) Fabris (Hircampuri, Hercampuri) as used for gastric secretion stimulant, anti-inflammatory (liver), blood purification, *Gentianella graminea* (H.B.K.) Fabris (Corpus huay, Chinchimali) for the stimulation of bile secretion, anti-inflammatory (liver), and blood purification, *Gentianella thyrsoidea* (Hook.) Fabris (Huallpa pachaqui, Japachanchara, Tucumia) for the stimulation of bile secretion, anti-inflammatory (liver), anti-diabetic and *Morus nigra* L. (Mora) as anthelmintic. Brack Egg [60], whole of Peru, mentions *Cheilanthes bonariensis* (Willd.) Proctor (Cuti Cuti) as febrifuge, *Gentiana sedifolia* HBK (Hercampure) as aphrodisiac; *Gentianella alborosea* (Gilg) Fabris (Hercampuri) for diabetes, as diuretic and to lower cholesterol; *Gentianella bicolor* (Wedd.) Pringle (Corpus huay, Chinchimali), *Gentianella thyrsoidea* (Hook.) Fabris (Corpus huay, Chinchimali) *Geranium dielsianum* Kunth (Pasuchaca) as anti-diabetics. Our own previous studies [33,61-70] all from Northern Peru, list *Pellaea ternifolia* C. Chr. (Cuti Cuti, Cute Cute, Cuticuti, Cute-Cute Amarillo, Cuti Cuti Amarillo) as used for diabetes and liver, *Gentianella alborosea* (Grimes) Pringle (Hercampuri) for diabetes; *Gentianella bicolor* (Wedd.) J. Pringle (Corpus Way, Corposhuar, Hornamo Leon) for arthritis, diabetes, bone pain, cholesterol, gastritis, liver, blood, rheumatism; *Gentianella bruneotincta* (Gilg.) J.S. Pringle. (Anga Macha) for Infections of the uterus, after giving birth; *Gentianella crassicaulis* J.S. Pringle (Violeta Genciana, Hojas de Violeta) for gastritis, “special” diabetes and dizziness; *Gentianella dianthoides* (H.B.K.) Fabris (Genciana, Egenciana, Amargon, Campanilla) for liver, kidneys, blood, as purgative to loosen the stomach, diabetes, cleansing, blood irrigation, blood problems, liver Infection; *Gentianella graminea* (H.B.K.) Fabris (Sumaran, Chinchimali, Corpushuay) for diabetes, liver, blood, burn fat, Intestinal fever, cough, fever, infection, allergies of the blood, varicose veins; *Geranium ayavacense* Willd ex H.B.K.*, Geranium sessiliflorum* Cavanilles (Puli Punchi, Pasuchaca, Pachuchaca, Miscamisca) and *Morus alba* L. (Morera) for diabetes; *Notholaena nivea* (Poir.) Desv. (Doradilla) and *Pellaea ternifolia* C. Chr. (Cuti Cuti, Cuti Cuti amarillo) against diabetes. All of the *Gentianella* species were used for psychosomatic disorders [71].

The present study confirms our initial hypothesis that at least in case of *Gentianella* and *Geranium* various species are sold under the same common names in Mercado Aviación in Lima, and in other parts of the country. The different fresh *Gentianella* species are readily identifiable botanically, but neither the collectors nor the vendors do make a direct distinction between species. However, at least half of the surveyed venders in Mercado Aviación sell *Gentianella* (Hercampuri) in finely powdered form, which makes the morphological identification of the species in the market impossible, and greatly increases the risk for the buyer. In case of *Geranium* (Pasuchaca) this is slightly less perilous, since none of the species appear to be toxic. However, they are mostly sold as dried rhizomes with few leaf fragments, and a morphological identification in the market is difficult even for trained botanists. The best way to ensure correct identification would be DNA bar-coding. The necessary technical infrastructure was however not available locally. The use of DNA bar-coding as quality control tool to verify species composition of samples on a large scale would require to carefully sample every batch of plant material sold in the market. The volatility of the markets make this is an impossible logistical task. We also confirmed that all of the species encountered, and the same or closely related species mentioned in literature sell under wide variety of common names. Worse, one species might be sold e.g. as “Hercampuri” in one location or market stand, while selling under a different name at a neighboring stand. As expected we did not find any consistency in the dosage of “Hercampuri” (ranging from 20–50 g/preparation) in Mercado Aviación, nor in the maximum number of days the preparation was to be taken (unlimited to a maximum of 15), nor in vendors agreeing on possible side effects. This is the more perilous as this stretched across species, i.e. *Gentianella thyrsoidea*, which clearly has toxic compounds, was often classified as benign, while other species of *Gentianella* were sold with clear warnings and vise versa. In case of “Pasuchaca” dosages were much more consistent, while the fern admixtures again varied wildly in dosage. The same holds true when comparing dosages with the use of the same or similar species mentioned in literature (Additional file 1). We also confirmed that the vendors in general knew what a plant was used for, that the material had to be boiled as tea, and which other ethnospecies were supposed to be included in a preparation, however, as indicated above, knowledge about side effects was sketchy.

Information on the possible efficacy of all the species researched is scarce. Karato et al. [72] report on some glucosidase inhibition in *Geranium dielsianum*, which might explain the use of *Geranium* in diabetes. However, *Geranium dielsianum* was hardly sold in Mercado Aviación. Some phytochemical analysis has been done on *Gentianella alborosea *[73], and the plant was shown to have free radical scavenging properties [74]. However, *Gentianella alborosea* again was not common in the market. *Gentianella nitida* has been a little better studies from a phytochemical perspective [75-77] and is also known for its antimicrobial and free radical scavenging activity [78]. None of these studies however confirm efficacy as anti-diabetic. Even worse, the botanical information of the species mentioned above is not free of doubt.

The only species in anti-diabetic mixtures encountered that actually has proven activity is the introduced *Morus alba *[79-93] This species is well known in Chinese Traditional Medicine, and also used other pharmacopoeiae, e.g. [94].

These results indicate that even in case of only a few plant species, used for a very clearly circumscribed application, patients run a considerable risk when purchasing their remedies in the market. The possible side effects in this case are the more serious because diabetes has to be treated long term, and as such the patients are ingesting possible toxic remedies over a long period of time. Much more control, and a much more stringent identification of the material sold in public markets, and entering the global supply chain via Internet sales, would be needed.

## Competing interest

The authors declare that they have no competing interest.

## Authors’ contributions

All authors participated in the fieldwork and literature research for this study. RB and NPZ analyzed the data and prepared the manuscript. All authors provided revisions to the manuscript. All authors read and approved the final manuscript.

## Supplementary Material

Additional file 1 Medicinal species found in the present study compared to literature. Click here for file
